# The Crisis of Health Professions Education in Pakistan

**DOI:** 10.15694/mep.2019.000027.1

**Published:** 2019-02-11

**Authors:** Arslaan Javaeed

**Affiliations:** 1 University of Ottawa

**Keywords:** health profession, education, crisis

## Abstract

This article was migrated. The article was marked as recommended.

Health professions are critical in maintaining the health infrastructure of the country. The quality of health care education imparted directly affects the people and it is thus very important to focus our attention towards it. This paper is focused on the current dilemmas being faced in the health professions education and a two-step solution was proposed in view of the deep understanding of the problem. The first step includes a teacher training course to educate and empower the educator about the current advancements in the healthcare professions education. The second step included a thorough evaluation of medical educators to assess their teaching practices and bring forward solutions to improve them. Undoubtedly it can be stated that there is a dire need to implement principles of health professions education in Pakistan and educators have to play their crucial role in understanding the current challenges and taking steps to overcome them.

## Introduction and Background

“What we want, and need is education pure and simple, and we shall make surer and faster progress when we devote ourselves to finding out just what education is and what conditions have to be satisfied in order that education may be a reality and not a name or slogan ( Dewey, 1938).”

The field of education has always fascinated me. It was during those long tedious hours in the lecture halls of my medical college that made me wonder whether there would be a better way to do this. The rote memorization, endless factual based assessments, lack of comprehension of content, didactic teaching and no interaction between the student and the educator still makes me dread medical school. Even after I graduated and started my post graduate degree in Histopathology, the scenario was similar, the ages of my colleagues and my professor being the only difference. I hoped that post graduate learning would be more comprehensive, concept based with focus on research and application of knowledge, better teaching methodology with utilization of technology and an interactive classroom but this soon turned out to be wishful thinking. Now as an Assistant Professor, standing in-front of hundreds of students, I was baffled about the sheer responsibility of teaching these naïve minds. What I saw were some brilliant minds waiting to be taught something new, something different. I did not want to demotivate them by going through what I went through, by repeating the same traditional methodology that my professors stuck with, and with this goal in mind, I enrolled in Master’s in health professions education.

My aspirations for this course were to bring me at a position to know how, in modern, fast-paced, technological advanced world, the vast subject of medical education is being dealt with. I was interested to learn what are the current approaches about content delivery, the theoretical background of teaching methodologies, the development of effective assessments, designing of appropriate curriculum so on and so forth. I wanted to learn the “arts and science” of helping students learn medicine. I wanted to be a learner again to deal with the inconsistencies of medical education that I faced during my medical journey and with the belief that only by educating myself, I could bring the change in my approach, practices and circle of influence.

My journey through Master’s in Health Professions Education program was a story in itself. The most important aspect for me was to learn how to critically analyze articles because as there is a lack of research in Pakistan, any article or book is taken as the supreme authority not to be challenged. This attitude was difficult to overcome, and it took me some time to analyze and critique on the suggested articles.

Through this course, I have developed a deeper understanding and compared and contrasted the ideas with what is being practiced currently in Pakistan. Having been taught there and now at a teaching post, I am in a unique position to state that health professions education is in a major crisis. Wherever I looked, I saw that aspect critically deficient. According to principles of andragogy ( Kaufmann, Mann & ASME, 2007), internal drive is one of the key factors in learning. In this paper I would argue that the internal drive of students is being crushed when going through an ill-conceived system of education. I would also argue about the dire need of health professions education in the country and why is it the responsibility of the educator to change the current deplorable condition of education.

Health professions education is gaining importance worldwide ( Gouda & Youssef, 2014). It is widely recognized as a mandatory specialty in medical schools to develop effective teaching strategies, mode of assessments, curriculum design and delivery of course content. Although Pakistan Medical and Dental Council (PMDC) recognizes the need to establish Department of Medical Education (DME) in every college, there is no emphasis on health professions education for medical educators. It is currently being offered in seven universities in Pakistan whereas the total number of medical colleges exceed 140. There is no concept of formal teaching courses or workshops for those who are going to teach the future doctors of the nation. A large number of doctors (approx. 9000) are being “manufactured” each year, but they lack the ability to analyze critically or self-reflect. Learning objectively through this course, has identified many gaps in the current education system which have been neglected, at best. The educators in medical colleges are not aware of new methods of teaching, theoretical background of their teaching practices, modern methods of assessments, the appropriate design of the curriculum and effective content delivery. There are similar problems in nursing education as well as post graduate residency training, but this will be out of the scope of this paper. For the ease of comprehension, the paper is divided into two major sections; the first section is dedicated to defining the problem i.e. overview of the conventional mode of medical education in Pakistan leading to crisis whereas the second section will delve into the steps proposed to resolve the crisis with an emphasis on central role of the educator.

## Conventional Mode of Medical Education

The system of medical education in Pakistan is undergoing a major crisis which needs urgent remedial measures. An in-depth analysis of the factors perpetuating this crisis is presented here. In this section, we will be discussing these factors such as outdated teaching methods, lack of research, ineffective assessment methods, poor curriculum design and substandard content delivery.

### Outdated Teaching Methodology

In undergraduate and post graduate studies, the mainstay is standardized form of teaching. Delivering a lecture is considered as the optimum source of transferring education to the students with minimum interaction. It would not be exaggerated to say that the teacher comes and delivers a lecture, prepared straight out of the book and reads it to an uninterested audience day after day. Any interruption in the form of questions or comments is considered as highly disrespectful. Almost seventy five percent of the course content is taught through this mode. The first two years are spent in this form of routine whereas clinical rotations start from 3 ^rd^ year. During clinical rounds, although there are patients, the preferred mode of teaching is still lecture. The highly busy educator will come, listen to two or three histories that the students have prepared, give a lecture on a relevant topic and leave. Even though concepts of situated learning can deeply engage and inspire a student, but the context is not utilized well by the educator due to his lack of knowledge of other teaching modalities. Sparapani & Perez (2015) argue that proponents of traditional teaching prefer the “order and stability” as opposed to “chaos and instability” in a classroom created by differential modes of instruction leading to complacence on the part of the educator and obstruction to learn on the part of the learner (p.83). It is unfortunate to see that “Education as transferring” and “Teaching as instructing” is still the major metaphor that would explain the teaching methodology with students being on the receiving end ( Davis, Sumara, & Luce-Kapler, 2015). The attitude of educators is one that of ignorance, because having done a degree in the relevant subject, they consider that they are competent enough to teach a class. Most of the teachers have not gone through any formal teaching methodology course and this reflects in their status quo practices of didactic teaching. The senior the teacher is, the stronger is his attitude of ignorance of the current needs of the millennial learner. It can be well assumed that if an educator is unware of the theories of learning (Kaufmann & Mann, 2014; Hodges & Kuper, 2012), how will he ever shatter his bubble of ignorance and construct an environment to facilitate learning of his students? There is no definite process involved as far as teaching methodology is concerned and because the teaching is not linked to any theoretical framework, there is no thought process involved as to how to make it better or what objectives will be gained through a particular mode of teaching. Although all of these are serious deficiencies ready to ruin the learning potential of the student but what is alarming is that the educators have become so complacent that don’t feel the need to bring any change and there is no relevant authority in Pakistan that would evaluate the competencies of the teacher or hold them accountable. Once hired at a public medical college, the teachers have secured a lofty position till the age of retirement, whether they teach effectively, or they do not.

Even the students are conditioned to believe that only if they take the traditional lecture, they will be able to understand the topic. Although a popular dictum across all medical colleges is that “no one is going to teach here, you have to learn yourself”, but somebody needs to facilitate how that learning is to be done. Kaufmann and Mann, 2014, state self-directed learning as “evidence of higher levels of individual development” which also collaborates with Maslow’s hierarchy (p.17) but I would argue that this should come internally rather than coerced externally. The learner should be provided with basic knowledge and then should have the opportunity to develop his own deep understanding. Dewey (1938) in his ground-breaking book “Education and Experience” states that “any experience is mis-educative that has the effect of arresting or distorting the growth of further experience” (p.25). This holds true for the medical education as the students instead of becoming life-long learners or passionate about learning, eventually just end up in “economic utility mode” of learning; to pass exams and to earn well through the degrees attained (Sparapani & Perez, p.84, 2015). The result of this form of teaching is that the students are not being engaged and their learning is not being enhanced. The topic stays within the confines of the four walls of the classroom and has no relevance in their daily lives although these are matters of routine healthcare.

### Substandard Content Delivery

Health Professions is one of the unique subjects where a lot of content is to be covered while keeping in mind the changing dynamics of healthcare professions. This requires the content to be delivered in effective ways for it to remain easy, relevant and understandable by the students. Technological advances such as online learning platforms, simulations, interactive learning applications, social media have taken the medical world by storm ( Cheston, Flickinger & Chisolm,2013), but educators are still lagging behind to accept it in formal ways of teaching. They rely on Power point presentations for most of the content with pictures scanned from the textbook. Use of videos, animations or social media is almost nonexistent. They are stuck with the “presentational view” of content delivery which relies on the availability of selective images and text which although can generate interest of the student but does not provide opportunity to interact or create something new (Anderson, p.37). It is a pity to imagine that such an interesting field of medical education, where there is plethora of available resources to develop the concepts and inspire the learner, is only left to be studied through a textbook, view a presentation and the imagination of the learner. The content delivery is so boring that the only reason the students attend classes is to maintain attendance of that particular subject as it will be counted towards the final grade. The learner as well as the educators are aware of this fact and 10,000 hours of prescribed teaching along the 5-year teaching period are just wasted in this drudging manner.

The future of medical education has long been taken outside the realm of a classroom where online teaching courses, online simulation skill labs, mobile applications, gaming and interactive teaching interfaces are being introduced to create a powerful learning experience ( Mehta, Hull, Young & Stoller, 2013; Gentry, Car, Car & Zary, 2016). The millennial using all these technologies in other aspects of life in the outside world and then coming to attend a power-point presentation in a classroom will not only feel disinterested but also disconnected. Keren-Kolb, 2013, argues that for a technology to be skillfully used for effective learning, it has to *engage* the learner by motivation and switching his role from a passive learner to an active one, *enhance* his learning by demonstrating it through unique means using the technology and *extend* his use of technology in his everyday life making him a lifelong learner i.e. “the triple E framework” that the educators must keep in mind while planning activities. As the students are constantly using their cell phones, the educators can manipulate the potential through the principles of triple-E and provide educational content through these means. Social media is increasingly being recognized as a great tool for medical education as content from all over the world can be viewed through Twitter, Facebook, Educational blogs etc. Although e-learning and use of social media is backed by learning theories ( Flynn, Jalali & Moreau, 2015) but these sources of knowledge are still considered as disruptive and shunned by the medical educators in Pakistan. During the entire 5-year period, the only time the student actually engages with the content is when the exams are due which is a gross failure on the part of the educator. Anderson argues that the modern education has to “support a journey towards capacity rather than competence” (p.42) so that the learners can deal with the challenges of the future. This current mode of medical education is nowhere near achieving this capacity in its students.

### Poor Curriculum Design

The MBBS curriculum, jointly prepared by the Pakistan Medical and Dental Council (PMDC) and Higher Education Commission, is a 120-page document, to be followed by all the medical colleges in the country. It is considered to be the guiding principle on which the curriculum of each medical college is to be based. As very few medical colleges have a department of medical education so the curriculum is implemented as it is with no changes. Going through the principles of curriculum development (Thomas & Kern, 2015) and then revisiting the PMDC curriculum, I found it to be rudimentary at best. The curriculum is deemed to be all the chapters of a text book in a sequential order. There is no working on why a specific topic should be included or excluded. Although medical education is one of the most complex programs, it is still presented in a narrative form with only emphasis on the learning objectives. How these learning objectives were derived or how are they to be achieved is not specified in the curriculum. Majority of the educators are unaware of critical concepts of Bloom’s taxonomy or Maslow’s hierarchy of needs so although the blanket term of “knowing the content” is used throughout the curriculum, it is not defined at what level. A general heading of teaching strategies is mentioned but does not specify when to be applied. The deplorable state is that its implementation and evaluation is not monitored so it is on the discretion of the educator to leave a particular topic or stress excessively. There is no accountability of completing the whole curriculum and much is left to the student to study himself. The topics deemed important from examination point of view are covered in detail whereas others, which might be more important in local or public health context, are left by the educators. Such discrepancies in developing and following the curriculum are in stark comparison to the process of curriculum design and development in the developed world of medical education (Thomas & Kern, 2015; Prideaux, 2003).

### Lack of Research

There is hardly any research that is taking place in the medical colleges although one expects it to be the hub of new ideas. This is partly due to the “follower mindset” of the educators rather than the “innovator”. One would argue that a country producing 9000 doctors annually should be at the forefront of research regarding educating health professionals but sadly that is not the case. The educators themselves are naïve of the research culture and cannot supervise or support the students in the process. There is no mandatory requirement to submit a research paper as part of the undergraduate curriculum so the students although interested, get demotivated by the lack of support they receive in this regard. As the educators are not identifying needs of the students, developing curriculum, improving teaching methodology or working on new methods of assessments, it is also not deemed as a significant exercise to spend time in research.

### Ineffective Assessment Methods

One of the key issues plaguing the undergraduate medical education is the ineffective methods of assessments. The programs are based on summative assessment at the end of the year which will render the student successful or otherwise. As the formative assessment throughout the year does not carry much weightage, students tend to work harder towards the end of the year rather than whole year round. The educators are often unaware of what to assess as their assessments are not based on any theoretical framework such as Bloom’s taxonomy or Miller’s hierarchy (Downing & Yudkowsky, p.4).The second issue is that they don’t know how to assess the content effectively as the strengths and weaknesses of each type of assessment such as multiple choice questions, short assay question, modified essay question, true/false, simulation etc. are not known in detail to them ( Epstein, 2007; Al-Wardy, 2010) Rote learning is still widely appreciated over understanding the content and is considered as hallmark of intelligence and hard work. Epstein, 2007, suggests that multiple methods of assessments can overcome the weaknesses of individual assessment and provide richer data to analyze a student’s progress (p.388). The majority of colleges follow multiple choice questions and short essay questions as their preferred mode of assessment for theory whereas objective structured practical examination (OSPE) and an oral exam is conducted for practical procedures. The construction of these assessment modes is not understood by most of the educators resulting in ineffective assessment methods. According to Van der Vleuten’s five criteria of utility of a particular assessment method (cited by Epstein, 2007, p.388), the assessments conducted in Pakistani medical colleges are not reliable, lack validity and do not have a significant impact on future learning.

## Role of Educator

**Figure 1.**
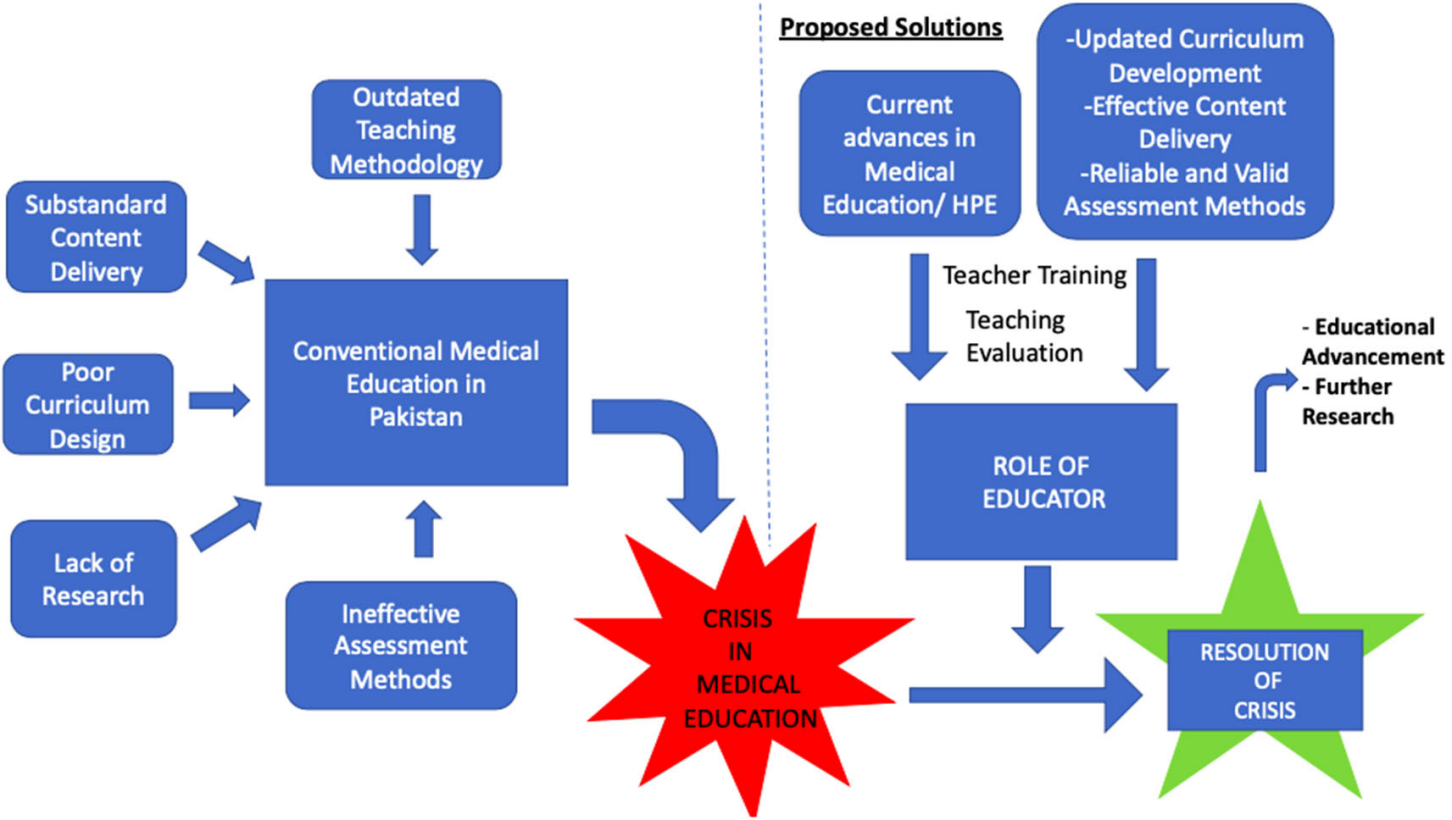
Framework highlighting the problems leading to crisis in medical education and proposed solutions for resolution of crisis.

In such critical situation, I would argue that there needs to be a sense of urgency in at least talking about these matters, realizing the gravity of it and taking appropriate actions to alleviate the situation. The educator’s responsibility in such circumstances would be manifold. There is work in every avenue to be done. He has to become the element of change by not only changing his attitude towards teaching but also learning actively about it. He needs to have a strong theoretical framework and have working knowledge of what teaching methodology is and how to incorporate it into his learning environment. He constantly needs to adapt his teaching style to suit the needs of his students. The system of assessment needs to be improved so that a specific structure is followed. A possible framework for tackling this grave issue is proposed in Figure 1.

As it is depicted in the framework, the role of educator lies at the centre to make a dramatic change from a crisis situation to a controlled one. It is only when the crisis has resolved that the medical educators in Pakistan can focus towards educational advancement and further research. Following is a brief description of the proposed 2- step process that would empower the educator in creating a difference.

### Step 1: Teacher Training Courses

Although PMDC has made it mandatory for all the medical colleges to establish a department of medical education, it is still at an age of infancy. As mentioned before, only few universities are offering master’s in health professions education so it will take a long time to have enough strength of competent educators. Till the system matures, PMDC should conduct regular teacher training courses by accomplished faculty, for all the educators to improve their teaching practices. I propose to include the educator while developing these courses so that valuable feedback is obtained, and special focus is given to the genuine needs of the educators rather than the perceived needs. The main aim of these courses would be to transform an educator from being a part of the problem to a part of the solution. This would be the most important step in creating awareness about the problem, create a need in the educators to find solutions to their teaching problems and create peer networks or “communities of practice” so that an ongoing process of transformation begins. These courses should be followed by refresher courses so that change in practice is ensured and new ideas are generated. Although it is difficult to address every issue, the teacher training courses can be developed on the key themes identified in the first section of the paper so that the grave issues are prioritised first.

a. Teaching Methodology:

An overview of all the teaching methodologies with hands-on practice should be emphasized. Theoretical framework behind each methodology and its implication in the health care education should be stressed upon so that educators can utilize these modalities with ease and conviction.

b. Standardized curriculum development:

The principles of curriculum development should be discussed in detail so that educators know how to make learning objectives according to Bloom’s taxonomy. Resources such as relevant articles, books etc. can be utilized to enhance this learning.

c. Effective use of technology in academic teaching:

The concept of technology as “distracting and useless” needs to be eradicated so that it can be utilized to its maximum potential. Different learning platforms on social media such as Twitter, Edmodo etc. should be shown as revolutionary in terms of delivering content, reaching all across the globe and generating interest with useful interaction. The concerns about these technologies should be actively sought and cleared with the help of current research.

d. Assessment methods:

A detailed discussion about different strategies of assessments, their strengths and weaknesses, and their applicability can be done in these courses so that educators are well aware of which modality to use to assess a specific content. Construction of these assessments is another important arena which needs on-going practice and feedback.

### Step 2: Teacher evaluation by competent authorities

As a proposed second step, all those teachers that have undergone the teacher training course should be evaluated by a competent authority to objectively assess if there are changes in the educational practices. A formative evaluation should be undertaken with feedback from the students as a guiding framework for further improvement. This form of individualized evaluation will help in identifying the strengths and weaknesses of the educator and will help in taking steps to improve them. The goal of this evaluation should not be punitive but to improve the existing practices of the educators. With this we come to the task of who should conduct this evaluation? The department of medical education, in each medical college, can be given the responsibility to undertake these evaluations along with an external review by PMDC.

## Future Goals

Through the above metioned steps, it is hoped that the educator will eventually be empowered to become the element of change. One may question why is the educator responsible for bringing all these changes? It can be stated that the educators’ responsibility is the basis of each solution as the burden of healthcare professional education lies on the shoulders of the educator till the system improves. This responsibility, if taken with a holistic view, can result in significant improvement in the current scenario. It will result in a workforce of individuals who will shape the future of health care professions in Pakistan.

## Take Home Messages

This paper is focused on the current dilemmas being faced in the health professions education and a two-step solution was proposed in view of the deep understanding of the problem. The first step includes a teacher training course to educate and empower the educator about the current advancements in the healthcare professions education. The second step included a thorough evaluation of medical educators to assess their teaching practices and bring forward solutions to improve them. Undoubtedly it can be stated that there is a dire need to implement principles of health professions education in Pakistan and educators have to play their crucial role in understanding the current challenges and taking steps to overcome them.

## Notes On Contributors

Dr. Arslaan Javaeed is an assistant professor of Pathology in Poonch Medical College, Rawalakot. This paper was written as an assignment for one of the courses taken towards the fulfilment of requirements of Masters in Health Profession Education program, from Faculty of Education, University of Ottawa, Canada.
