# Supplementary material for: The Crisis of Health Professions Education in Pakistan
Source: MedEdPublish (2016). 2019 Feb 11;8:27. [Version 1] doi: 10.15694/mep.2019.000027.1 (PMC10712578; doi:10.15694/mep.2019.000027.1)
Supplement: Peer review history

# Reviewer report: Reviewer response for version 1

**DOI:** 10.21956/mep.19772.r29699

**Published:** 2019-02-20

**Authors:** P Ravi Shankar

**Affiliations:** 1 American International Medical University

This review has been migrated. The reviewer awarded 4 stars out of 5 Thank you for the invitation to review this interesting paper. The paper presents an overview of the present situation of medical education in Pakistan. Here in the Caribbean like in Pakistan, lectures are the predominant learning method. At a medical school in Aruba I was partly successful in introducing other teaching-learning methodologies. I am surprised that even Deans and Associate Deans at medical schools are only familiar with lectures and are not aware about other methodologies. Even the term ‘lecturer’ used for faculty members at many institutions highlights the total dominance of lectures. As a faculty all you are expected to do is lecture. The ability to critically analyze and interpret information and develop self-directed learning skills is important for medical students. Many teachers view technology as disruptive rather than as enhancing learning. Many teachers ban mobile phones and tablets from their sessions altogether. I would argue that such a decision underscores their lack of knowledge of the power and impact of these technologies. All technologies have the potential to be used or misused. Also in South Asia the teacher is on a pedestal and questions from students and alternate points of view are not always welcomed. In South Asia, the regulators have a penchant for imposing a central curriculum and methods of teaching-learning on all medical schools. In most developed nations only the required competencies are defined and a model curriculum may be framed. The FAIMER institutes in India have led to the creation of a large pool of educators. There are positive developments in the field of medical education though many challenges still remain. Assessment drives learning and a proper understanding of different assessment methods is critical. I enjoyed the figure provided by the author which succinctly sums up the main arguments in the paper. The quality of written English is good though in a few locations corrections may further strengthen the paper. This article would be of broad interest to all medical educators.

Reviewer Expertise:

NA

No decision status is available

# Reviewer report: Reviewer response for version 1

**DOI:** 10.21956/mep.19772.r29700

**Published:** 2019-02-12

**Authors:** BALAJI ARUMUGAM

**Affiliations:** 1 TAGORE MEDICAL COLLEGE AND HOSPITAL

This review has been migrated. The reviewer awarded 3 stars out of 5 The article was nicely written. The article demonstrates the importance of the improvements in the medical education system that exists in the author’s country. The picture clearly expresses the view of conventional medical education and its problems, the proposed solutions like updating the curriculum, content delivery (Proper Teaching learning methods) and the appropriate assessment methods. There are lot of changes and updates in the medical curriculum recently in India. The changes are introduction of Competency based medical curriculum document 2017 to be implemented by 2019. As the author represented the two steps, the revolutionary approach towards the implementation of CBME started with CISP training at nodal centers followed by TOTs at their medical colleges under supervision is undertaken slowly. I congratulate the author for his personal view on the current scenario and the solutions for the changes in the medical education system.

Reviewer Expertise:

NA

No decision status is available

# Reviewer report: Reviewer response for version 1

**DOI:** 10.21956/mep.19772.r29701

**Published:** 2019-02-11

**Authors:** Trevor Gibbs

**Affiliations:** 1 AMEE

This review has been migrated. The reviewer awarded 3 stars out of 5 Thank you for asking me to review this opinion piece. The paper is well written and clearly demonstrates the passion from the author about medical education in the country of their birth ( I presume). Although the paper is specific about Pakistan, I would say that, rather sadly, it also reflects medical education in other resource-constrained and still-developing countries. The problems are clearly outlined as are the possible solutions, but I feel that what was missing was some possible practical solutions- how can we or how can we help in applying those modern methods of education to a country that desperately needs an effective and efficient healthcare system. It seems the author has found hos own way through travelling and possibly residing in Canada to complete his Masters course- could this be extended to others; how can we change the fixed mindset of those in power and with influence; how can the medical community as a whole help.An interesting paper to read but one that without these suggestions / potential activities might still fall on "silent hearing"

Reviewer Expertise:

NA

No decision status is available
